# Enhanced Ionic Polymer–Metal Composites with Nanocomposite Electrodes for Restoring Eyelid Movement of Patients with Ptosis

**DOI:** 10.3390/nano13030473

**Published:** 2023-01-24

**Authors:** Sara Sadat Hosseini, Bakhtiar Yamini, Levan Ichkitidze, Majid Asadi, Julie Fernandez, Seifollah Gholampour

**Affiliations:** 1 Department of Neurological Surgery, University of Chicago, Chicago, IL 60637, USA; 2 Institute of Biomedical Systems of National Research University of Electronic Technology (MIET), 124498 Moscow, Russia; 3 Institute of Bionic Technologies and Engineering of I.M. Sechenov First Moscow State Medical University, 119991 Moscow, Russia; 4 Northern Michigan University, Marquette, MI 49855, USA

**Keywords:** ptosis, artificial muscle, ionic polymer–metal composites (IPMC), carbon nanotube (CNT), bovine serum albumin (BSA), microcrystalline cellulose (MCC)

## Abstract

The present study aims to use enhanced ionic polymer–metal composites (IPMC) as an artificial muscle (a soft-active actuator) to restore eyelid movement of patients with ptosis. The previous eyelid movement mechanisms contained drawbacks, specifically in the lower eyelid. We used finite element analysis (FEA) to find the optimal mechanism among two different models (A and B). In addition to common electrodes of IPMC (gold and platinum), the bovine serum albumin (BSA) and microcrystalline cellulose (MCC) polymers, with optimal weight percentages of carbon nanotube (CNT) nanofiller, were also utilized as non-metallic electrodes to improve the efficiency of the IPMC actuator. In both models, IPMC with nanocomposite electrodes had higher efficiency as compared to the metallic electrodes. In model A, which moved eyelids indirectly, IPMC with MCC-CNT electrode generated a higher force (25.4%) and less stress (5.9 times) as compared to IPMC with BSA-CNT electrode. However, the use of model A (even with IPMCs) with nanocomposite electrodes can have limitations such as possible malposition issues in the eyelids (especially lower). IPMC with MCC-CNT nanocomposite electrode under model B, which moved eyelids directly, was the most efficient option to restore eyelid movement. It led to higher displacements and lower mechanical stress damage as compared to the BSA-CNT. This finding may provide surgeons with valuable data to open a window in the treatment of patients with ptosis.

## 1. Introduction

A measure of 0.3% of American and Western European people suffer from facial nerve palsy (FNP) every year [1,2]. FNP and Bell palsy can lead to ptosis, and consequently, loss of eyelid movement, blinking disruption, and corneal damage. Various central nervous system disorders may also lead to ptosis and eyelid dysfunction [3,4,5,6,7]. Eye blinking is typically performed by the muscles of the orbicularis oculi and levator, which are responsible for closing and opening the eyelid, respectively [8]. Several nerve and muscle transplants have been performed to restore eyelid movement [9]. Anterior and posterior surgical approaches over the levator palpebrae and Muller muscle are also two common methods to treat these patients [10]. However, the efficiency of the current treatment methods is controversial and challenging, and there is not an exact surgery treatment for these patients [9,10].

A gold or platinum implant can be used to close the upper eyelids and protect the corneas. These implants are passive and cannot necessarily open and close eyelids to blink independently. Kozaki et al. suggested a wearable robot with rigid actuators to support eyelid movement in patients with ptosis [11]. Wearable robots are not user-friendly models due to their inadequate flexibility and high weight [12]. Hesmat et al. used an electromagnetic actuator as an artificial muscle to evaluate the optimized forces and positions for efficient indirect movement of eyelids using a sling [13]. Mazza et al. suggested shape memory alloy (SMA) as an active artificial muscle to restore eyelid movement [14]. They tried to optimize the eyelid reflection and power consumption of SMAs. The studies by Ledgerwood et al. were helpful in the development of this field [9]. They showed the efficiency of electroactive polymer (EAP) with the metal electrode as an artificial muscle to treat patients with ptosis for indirect movement of the eyelids through a sling. The efficiency of their mechanism, specifically in the lower eyelid, contained drawbacks.

In the present study, ionic polymer–metal composite (IPMC), one of the most optimal and novel biological classes of the EAPs [15,16,17,18,19,20], was used as an artificial muscle (a soft-active actuator) with two different mechanisms to restore eyelid movement following ptosis. IPMCs have higher repeatability and more biocompatibility with lower required voltage as compared to other EAP classes for working in a wet biological condition [21,22]. The present study tried to find an optimal active model for IPMC to alleviate the drawbacks of the previous mechanisms in eyelid movement. One of the most important concerns in biological applications of IPMCs is the use of metals (i.e., gold and platinum) as their electrodes. We also aim to suggest the most efficient biocompatible non-metallic nanocomposites among bovine serum albumin (BSA) and microcrystalline cellulose (MCC) polymers with carbon nanotube (CNT) nanofillers as the alternative for metallic electrodes.

## 2. Materials and Methods

### 2.1. Study Design

**Figure 1:**
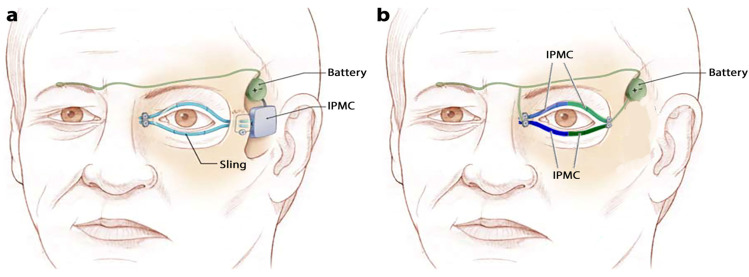
(**a**,**b**) show the schematic mechanisms of models A and B, respectively. Original (panel (**a**)) and modified (panel (**b**)) images were used with permission from the work of Ledgerwood and Tollefson et al., “Artificial Muscle for Reanimation of the Paralyzed Face” Arch Facial Plast Surg. 2012; 14(6):413–418) [9].

We used enhanced IPMC with nanocomposite electrodes as an actuator under two different mechanisms including models A and B to restore eyelid movement of patients with ptosis (Figure 1a,b). The importance and necessity of force and displacement in eyelid movement evaluation were shown in previous studies [11,13,14,15]. COMSOL Multiphysics (version 5.3; COMSOL, Inc., Burlington, MA, USA) was used for geometrical modeling, mesh generation, and finite element analysis (FEA) to calculate output forces, displacements, von Mises stresses, and cation concentrations in IPMCs. FEA-based simulation is common to solve biological problems [23,24,25]. The transport of diluted species and general form partsssial differential equations were used to define Nernst–Planck and Poisson equations, respectively, during FE simulation of IPMCs. The above modules were defined in the membrane (Nafion layer) of IPMCs in models’ A and B. Electric currents and solid mechanic modules were also used to simulate the electrodes and mechanical properties of the IPMCs. The selected non-metallic nanocomposite electrodes for IPMCs include BSA and MCC polymer matrices, which were combined with the optimum weight percentages of CNT nanofillers. It should be mentioned that the biological application and biocompatibility of BSA and MCC polymers were confirmed in a previous study [26]. Previous studies also confirmed that CNT is one of the most optimal nanofillers when used with polymer matrices in biological applications [27,28,29,30,31,32,33,34,35,36,37,38,39]. In addition to using the enhanced IPMC with BSA-CNT and MCC-CNT electrodes, we also used the IPMC with common electrodes (platinum and gold) for data validation and comparing the efficiency of enhanced IPMCs and common IPMCs.

The iconic current in the polymer is calculated based on the Nernst–Plank equation (Equation (1)) [40]. Equation (2) is used as the mobility equation and Equation (4) expresses the ρ (charge density). It should be noted that the electric potential was calculated by Equations (3) (Poisson’s equation) and (4) [40].

(1) $$\frac{\partial C}{\partial t}+\nabla \cdot \left(-D\nabla C-Z\mu FC\nabla \emptyset -\mu C\nabla \emptyset -\mu C\Delta V\nabla P\right)=0$$

(2) $$\mu =\frac{D}{RT}$$

(3) $$-\nabla \emptyset =\frac{\rho }{\varepsilon }\;$$

(4) $$\rho =F\left(C-C_{0}\right)\;$$

where *C* is cation concentration, μ is the mobility of cations, ΔV is the molar that quantifies the cation hydrophilicity, P is solvent pressure, and ∅ is the electrical potential in the polymer. It should be noted that D (diffusion), R (gas constant), T (temperature), and Z (ion capacity) equal 7 × 10–11 m^2^/s, 8.31 J/mol·K, 293 K, and 1, respectively [40]. The constant values for Ɛ, F (Faraday constant), and $C_{0}$ (initial cation concentration) were defined 2 m·F/m, 96485.34 s·A/mol, and 1200 mol/m^3^, respectively [40]. Material properties of the IPMC substructures are listed in Table 1 [26,27,28,29,30,41,42,43].

### 2.2. Design of Models A and B

**Figure 2:**
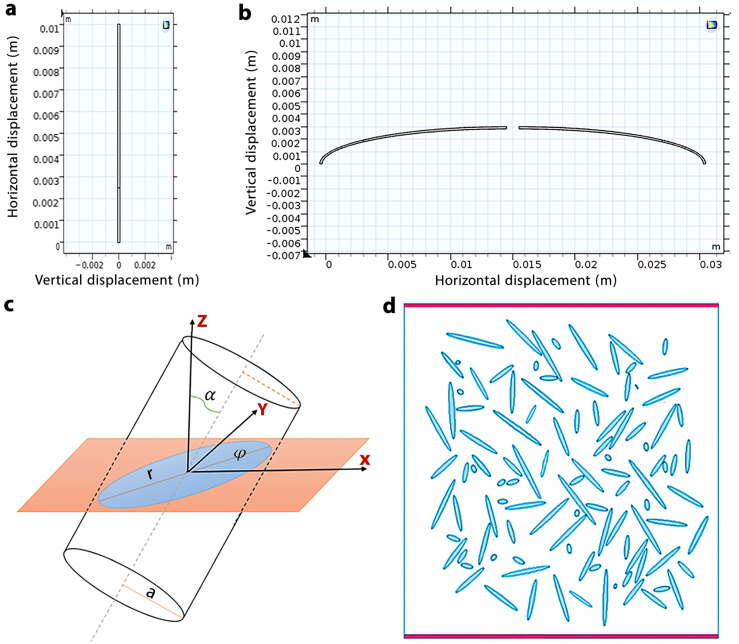
(**a**,**b**) show the IPMC strips designed for models A and B, respectively. (**c**) Spatial distribution of CNT on its elliptical cross-section. (**d**) The cross-section of a nanocomposite CNT based and distribution of CNT.

The IPMC strip in model A was designed for implantation in the temporal fossa region (Figure 1a and Figure 2a). In this model, similar to previous studies [9,15,16], we focused on implanting an expanded polytetrafluoroethylene sling to indirectly open/close the eyelids, rather than attempting to stimulate the existing muscles. This means that the force generated by IPMC in model A will move the eyelid levator muscle via the sling (Figure 1a). According to the study by Ofir et al. [44], the normal physiological force of the levator and frontalis muscles for blinking is 0.37–0.55 N. Hence, we used inlet voltage that equaled 3.2 sin(πt/4) to generate the forces in the normal physiological range in IPMCs with nanocomposite electrodes.

Based on the actual curvature shape of the eyelid, two curvature IPMC strips were designed in model B for direct movement of the eyelids without needing a sling (Figure 1b and Figure 2b). The general pattern and mechanisms of model B were inspired by the mechanism of wearable robots in a study by Kozaki et al. and direct eyelid movement by an SMA actuator in a study by Mazza et al. [11,14]. The inlet voltage in model B was able to produce linear displacement in the normal physiological range (3–6.5 mm [15]) in IPMCs with nanocomposite electrodes was 0.4sin(πt/4). IPMC displacements lead to eyelid movement in model B, hence, the effective output in the evaluation of model B is displacement, contrary to model A which was a force. It is worth mentioning that the difference between amplitudes of inlet voltages of model A and B is related to differences in their mechanisms as well as their methods to produce natural force (in model A) and linear displacement (in model B) in IPMCs to move the eyelids.

### 2.3. Simulation Methodology

#### 2.3.1. Percolation Theory 

The electrical percolation threshold (EPT) and consequently the optimal weight percentage of the CNTs in the BSA and MCC matrices are the most important material properties of the nanocomposites. The optimum values of the weight percentages were obtained from the random distribution of the CNT fillers in the matrices using MATLAB software (version R2018; Mathworks, Natick, MA, USA). It should be noted that the amount of electric conductivity at this optimum weight percentage was used as the optimum electric conductivity of the nanocomposite during simulation by COMSOL software.

To obtain the electric conductivity, the calculation of the parameter titled the EPT is required. CNTs combined with polymers at a particular weight fraction called the EPT result in several orders of magnitude increase in the electrical conductivity of the polymer–CNT nanocomposite. Uniform distribution of the CNTs throughout the polymer matrix will increase the probability of the percolation phenomenon due to the predominance of the conduction region in the dielectric and increase the current density. In the percolation region, the flow of electric charges in a dielectric will occur through the connection of CNTs to each other, which is done by quantum tunneling or hopping transport of the electrons between nanotubes that are close together [45,46,47,48]. In order to calculate the EPT and the electric conductivity of the nanocomposites, two steps were considered:

In the first step, a model was designed using COMSOL software in which 200 CNTs were randomly distributed as fillers in the cube-shaped geometry of the BSA and MCC matrices. The purpose of this new modeling was to simulate the EPT values of the nanocomposites, and to estimate the relative change in current density J_0_/(J − J_0_). In this simulation, a homogeneous dispersion state of the inclusions into the BSA and MCC matrices was considered using the excluded volume approach (Vex) [46]. According to Figure 2c, the excluded volume Vex is the volume surrounding a particle in such a way that the center of a similar particle in its vicinity is not allowed to penetrate it, so that it does not overlap with its neighboring particles. This method is based on the fact that the EPT is related to Vex rather than the actual volume of the filler particles.

Figure 2d shows a spatial distribution of CNTs and their elliptical cross-section. To consider the proper aspect ratio is to assume that CNTs are randomly distributed uniformly in a three-dimensional space. As can be seen from Figure 2c, due to the direction of cutting the nanotubes, their cross-section is exhibited as an elliptical form. In some cases, when r and a pertinent to the semi-major and semi-minor axes of an ellipse, are equal, the cross-section of the CNT will appear as a circle [45,46,47,48].

**Figure 3:**
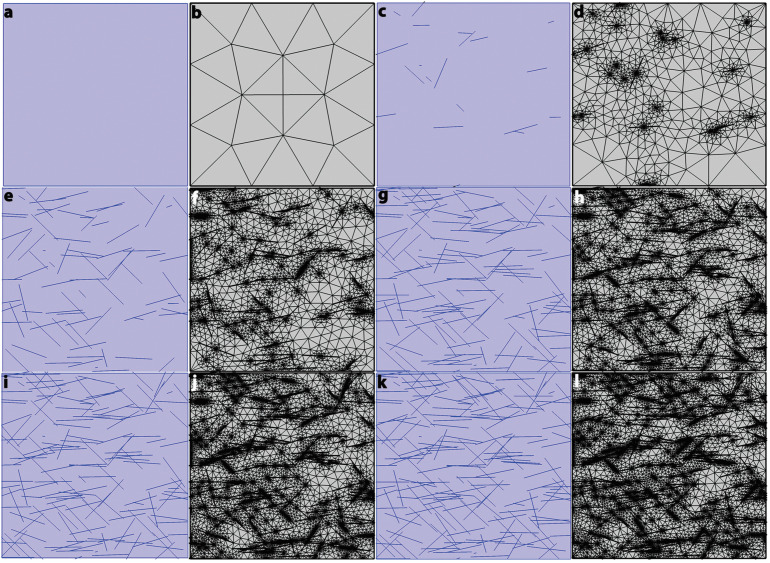
(**a,b**) show the random distribution of 0 CNT in the basic polymer and meshed basic polymer, respectively. (**c**,**d**) show the random distribution of 20 CNT in the basic polymer and meshed basic polymer, respectively. (**e**,**f**) show the random distribution of 80 CNT in the basic polymer and meshed basic polymer, respectively. (**g**,**h**) show the random distribution of 100 CNT in the basic polymer and meshed basic polymer, respectively. (**i**,**j**) show the random distribution of 160 CNT in the basic polymer and meshed basic polymer, respectively. (**k**,**l**) show the random distribution of 190 CNT in the basic polymer and meshed basic polymer, respectively. It should be noted that “20 CNT” means there is a 20 CNT particle in the basic polymer.

In the second step, random numbers were generated by MATLAB software. It should be noted that these random numbers are used for the x and y coordinates of the position of the CNT’s centers, which are distributed in the polymer based on optimal diameter and length (Figure 2c). In determining the geometric parameters of an ellipse, according to Equations (5)–(9), the random function is used:

(5) y=[(w−2r)×Rand]+r

(6) x=[(l−2r)×Rand]+r

(7) $$\alpha =\left(\frac{pi}{2}\right)\times Rand$$

(8) $$\alpha =\left(\frac{pi}{2}\right)\times Rand$$

(9) $$r=\frac{a}{\cos\theta }$$

where *x* and *y* are the coordinates of the center of the ellipse, l and w are the thickness and width of the composite (l = w = 10 µm), α is the polar angle, and φ is the azimuth angle of the CNTs in space. An exhibition of the final model design is given in Figure 3, in which CNTs are dispersed randomly in the BSA and MCC matrices considering the numbers of CNT as 0, 20, 80, 100, 160, and 190.

#### 2.3.2. Calculation of the Electrical Conductivity and Permittivity

The material properties of BSA, MCC, and CNT including electric conductivity, permittivity, and density are listed in Table 1. The electrical conductivity and permittivity of both nanocomposite electrodes were calculated as follows.

#### 2.3.3. Calculation of the Electrical Conductivity Using Bruggeman’s Symmetric Equation 

Bruggeman’s symmetric equation (Equation (10)) is derived from the effective medium approximation and percolation theory, which applies to heterogeneous mediums with different electrical properties. For a polymer–CNT composite with effective electric conductivity of σeff, this model includes a particular state in which s and t are equal to 1:

(10) $$\frac{\left(1-\Phi \right)\left(\sigma_{m}-\sigma_{\mathrm{eff}}\right)}{\left(\sigma_{m}+\sigma_{\mathrm{eff}}\right)}+\frac{\Phi \left(\sigma_{i}-\sigma_{\mathrm{eff}}\right)}{\left(\sigma_{i}+\sigma_{\mathrm{eff}}\right)}=0$$

where Φ as the surface or volume fraction of the CNTs, σm, and σi are the electric conductivity of the polymer matrix and CNTs, respectively [49].

Adding the CNTs to a polymer matrix leads to a change in the dielectric constant of the polymer material. If the alignment dispersion state of the CNTs would be considered, the capacitive region will dominate the entire dielectric volume, which will increase the effective electrical conductivity of the BSA–CNT and MCC–CNT composites. The uniform distribution of CNTs in the polymer matrix (due to the increase in the probability of the percolation phenomenon) will lead to the dominance of the resistive region in the dielectric and increase the current density. In the resistive region, the electric charge will flow in the dielectric, and connect the CNTs with each other, which is done through quantum tunneling or hopping of the electrons between CNTs that are close to each other. The dielectric constant of a polymer-CNT composite is calculated using one of the models of percolation theory, known as effective environment theory.

#### 2.3.4. Calculation of the Permittivity Using the Maxwell–Garnett Model 

For the determination of the effective permittivity of a composite, several models are considered, including Maxwell–Garnet (MG-2D) and Brugman symmetric models [49]. In this study, the Maxwell–Garnett model is used to calculate the effective permittivity of the composites. This theory is based on the electric polarization created by the application of a uniform electric field on the composite that predicts the electrical properties of a heterogeneous medium. According to this model, CNTs are simulated as infinitely long cylinders in two dimensions; thus, the electric field is perpendicular to the central axis of these cylinders. In the two-dimensional state, the effective permittivity of a composite, namely εeff, is calculated by Equation (11):

(11) $$\varepsilon_{eff}=\varepsilon_{m}+2\emptyset \varepsilon_{m}\frac{\varepsilon_{i}-\varepsilon_{m}}{\varepsilon_{i}+\varepsilon_{m}-\emptyset \left(\varepsilon_{i}-\varepsilon_{m}\right)}$$

where ∅ is the volume or surface fraction of CNTs, $\varepsilon_{m}$ and $\varepsilon_{i}$ are the electrical permittivity of the polymer matrix and CNT’s inclusions, respectively [50].

#### 2.3.5. Grid Independence Study 

**Figure 4:**
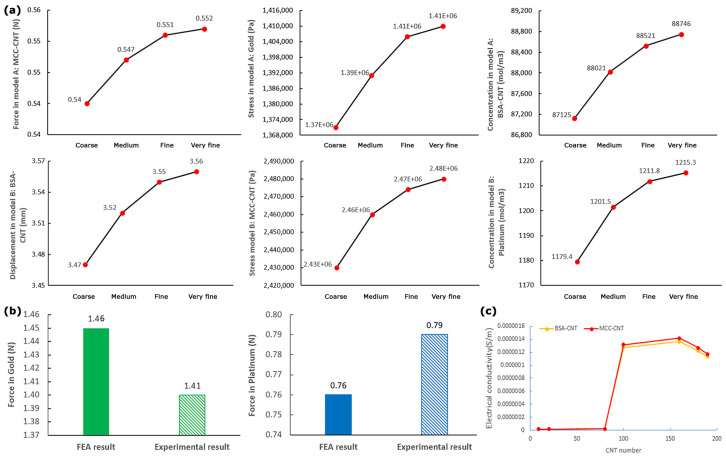
(**a**) Grid independence study for force, linear displacement, cation concentration, and stress values. (**b**) Comparison of FEA and experimental force results. (**c**) Electrical conductivity in BSA-CNT and MCC-CNT nanocomposites in regard to the CNT numbers.

The number of elements that were applied in COMSOL software for the meshing of models A and B were 21,944 and 39,433, respectively. The time step that was used in the simulation process was 0.1. The effect of mesh size on FEA results was evaluated using the grid independence test. The maximum difference in output values (force, displacements, stress, and cation concentration) in fine and very fine meshes for all models were less than 0.28% (Figure 4a). The smaller step sizes did not lead to differences in the results. The results of Figure 4a can confirm the correctness of the mesh convergence process.

## 3. Results

### 3.1. Data Validation

**Figure 5:**
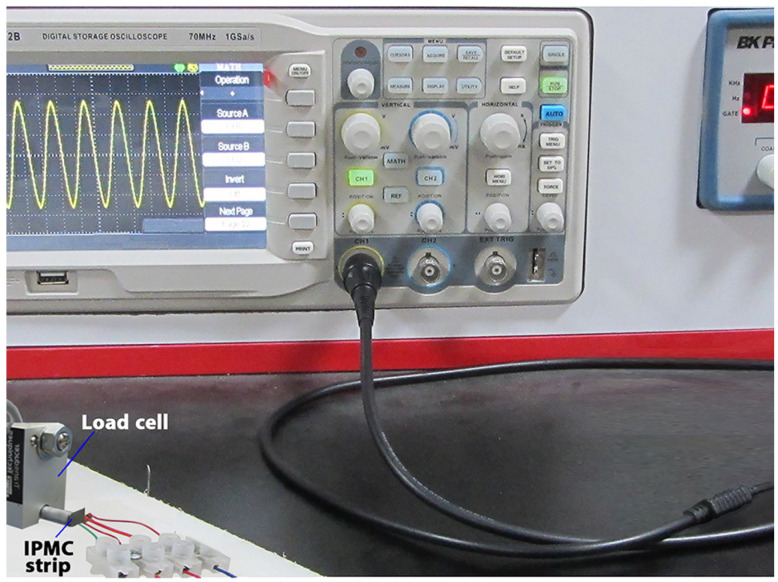
Devices for experimental force measurement.

After reviewing the grid independence study, an experimental test (Figure 5) was performed to validate the correctness of the force results. In regard to our limitations, we compared the experimental and FEA results for force in the tip of IPMC with common electrodes (gold and platinum) in Figure 4b. The results of Figure 4b showed that the differences between the experimental and FEA results of force were less than 3.8%. It should be noted that we repeated each experimental force measurement (by GSO-10 load cell) five times to ensure the reliability of the experimental results. 

### 3.2. Optimal Electrical Conductivity

The optimal electrical conductivities of BSA-CNT and MCC-CNT electrodes were calculated based on the EPT values and were used as an inlet in COMSOL software for FEA. The optimal electrical conductivity of BSA-CNT and MCC-CNT equaled 1.27 × 10^−6^ S/m and 1.31 × 10^−6^ S/m, respectively (Figure 4c, Figures S1 and S2). The corresponding values for gold and platinum electrodes had been defined as default values in COMSOL software as 4.44 × 107 S/m and 8.74 × 106 S/m, respectively.

### 3.3. Evaluation of Model A 

**Figure 6:**
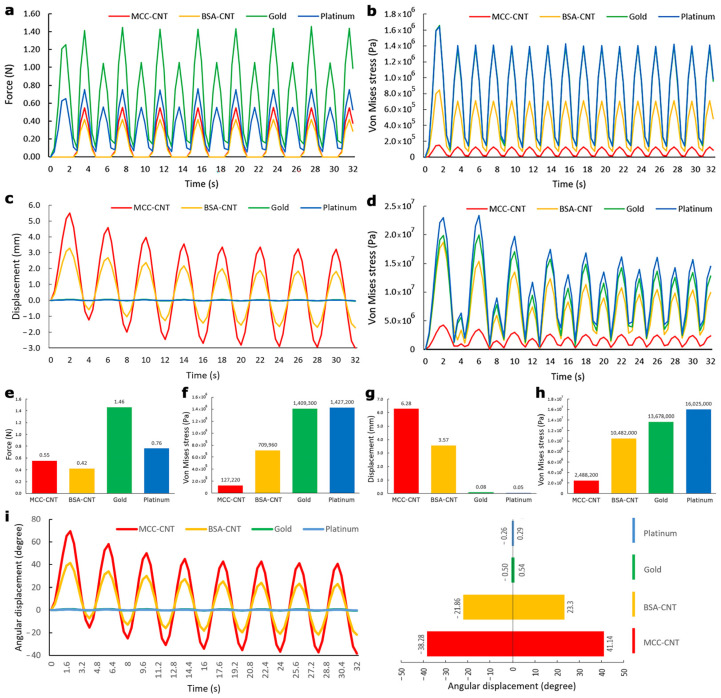
(**a**,**b**) show output force and von Mises stress in IPMC with all electrodes for model A, respectively. (**c**,**d**) show linear displacement and von Mises stress in IPMC with all electrodes for model B, respectively. (**e**,**f**) show the maximum force and von Mises stress in IPMC with all electrodes for model A, respectively. (**g**,**h**) show the maximum linear displacement and von Mises stress in IPMC with all electrodes for model B, respectively. (**i**) shows angular displacemsents in IPMC with metal and nanocomposite electrodes.

The main results for model A include force, stress, and cation concentration that were indirectly (via a sling) responsible to open and close the eyelids (Figure 6a,b,e,f). The sinusoidal results in model A reached the stable condition after 4 s, then the values were reported.

The generated forces in IPMCs with four electrodes were shown in Figure 6a,e. The lowest generated force among IPMCs with all nanocomposite and metallic electrodes belonged to IPMC with BSA-CNT nanocomposite (0.41 N). This value was 46.0% less than the lowest generated forces in IPMCs with metallic electrodes (platinum (0.76 N)). This value was also 25.4% less than generated force in IPMC with MCC-CNT nanocomposite (0.55 N). It should be noted that the generated force in model A of the study by Hasmat et al. with an electromagnetic actuator [13] was 0.65 N and Senders et al. with an EPAM actuator [15] was 0.63–1.5 N, respectively.

**Figure 7:**
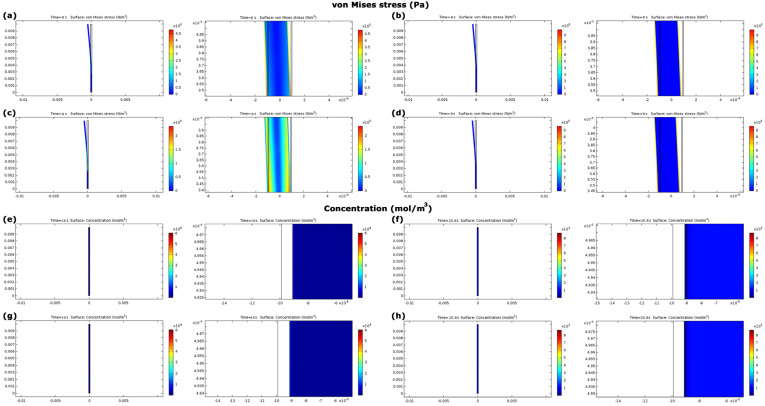
(**a**–**d**) show the distribution of von Mises stress in IPMC with BSA–CNT, gold, MCC–CNT, and platinum electrodes with their zoom panels, respectively. (**e**–**h**) show the distributions of cation concentrations in IPMC with BSA–CNT, gold, MCC–CNT, and platinum electrodes with their zoom panels, respectively. The panels are not to scale.

The lowest von Mises stresses in metallic and nanocomposite electrodes belonged to gold (14.1 × 105 Pa) and MCC–CNT (1.2 × 105 Pa), respectively (Figure 6b,f). The stress values in IPMC with nanocomposite electrodes were at least 49.6% less than those of metallic electrodes (Figure 6b,f and Figure 7a–d). This trend was also established for the force values. In IPMCs with nanocomposite electrodes, the stress in IPMC with MCC-CNT electrode was 83.0% less than BSA-CNT.

**Figure 8:**
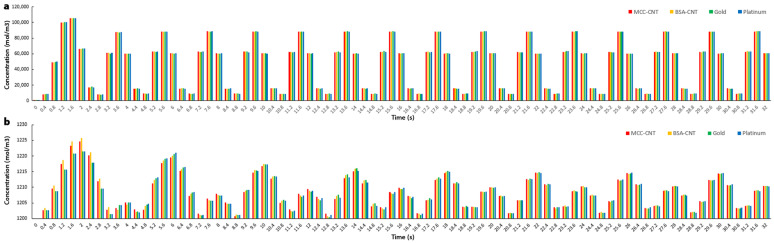
(**a**,**b**) show cation concentrations in different seconds in IPMC with several electrodes in models A and B, respectively.

Figure 7e–h and Figure 8a showed the cation concentrations in various seconds. The results of Figure 8a showed that the cation concentrations in IPMC with different electrodes under model A were almost the same with an error of less than 1.0%.

### 3.4. Evaluation of Model B 

The main results for model B include linear and angular displacements, stress, and cation concentration, which this model can be directly responsible to open and close the eyelids (Figure 6c,d,g,h). The sinusoidal results of model B reached stable condition after 24 s (Figure 6c,i), then the values were reported.

**Figure 9:**
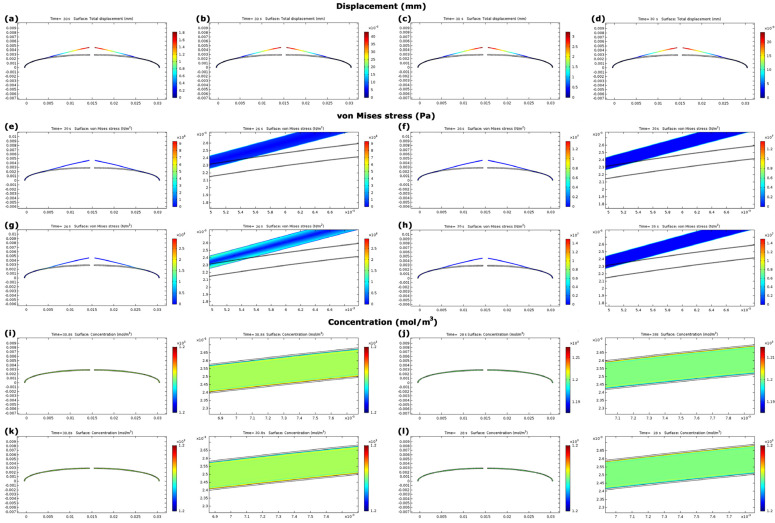
(**a**–**d**) show the linear displacement distributions in IPMC with BSA–CNT, gold, MCC–CNT, and platinum electrodes, respectively. (**e**–**h**) show the distributions of von Mises stress in IPMC with BSA–CNT, gold, MCC–CNT, and platinum electrodes with their zoom panels, respectively. (**i**–**l**) show the distributions of cation concentration in IPMC with BSA–CNT, gold, MCC–CNT, and platinum electrodes with their zoom panels, respectively. The panels are not to scale.

Maximum linear displacements in IPMC with MCC–CNT, BSA–CNT, gold, and platinum electrodes were 6.2 mm, 3.5 mm, 0.08 mm, and 0.04 mm, respectively (Figure 6c,g and Figure 9a–d). This means that the maximum displacements in IPMC with nanocomposite electrodes were at least 43.7 times higher than those of metallic electrodes. Among nanocomposite electrodes, the linear displacement of IPMC with MCC-CNT electrode was 77.0% higher than that of IPMC with BSA-CNT electrodes (Figure 6c,g). The angular displacements in IPMC with MCC-CNT were −38.28° to 41.14°, BSA-CNT were −21.86° to 23.30°, for gold were 0.50° to 0.54°, and for platinum were −0.26° to 0.29°, respectively (Figure 6i). The range of angular displacement in IPMC with nanocomposite electrodes (specifically MCC–CNT electrode) was higher than in metal electrodes.

The minimum von Mises stresses in nanocomposite and metallic electrodes were in IPMC with MCC–CNT (0.24 × 10^7^ Pa) and gold (1.37 × 10^7^ Pa), respectively (Figure 6d,h). The stress in IPMCs with nanocomposite electrodes was at least 23.3% less than in metallic electrodes (Figure 6d,h and Figure 9e–h). In nanocomposite electrodes, the stress in IPMC with MCC–CNT was 77.1% less than in IPMC with BSA–CNT electrodes.

The cation concentrations in IPMC with different electrodes began from the initial value of 1200 mol/m^3^. The cation concentrations had various values at different times; however, their changes in IPMC with four different electrodes under model B were less than 1.0% (Figure 8b and Figure 9i–l), similar to model A.

## 4. Discussion

Restoring the movement of the paralyzed eyelid is of great importance. This can preserve vision, decrease corneal exposure, and improve appearance. The present study aimed at the use of IPMC as an artificial muscle (a soft-active actuator) under the two different mechanisms (models A and B) to restore eyelid movements in patients who suffered from eyelid dysfunction and ptosis. IPMCs in both models have been enhanced by two non-metallic nanocomposite electrodes including BSA–CNT and MCC–CNT.

Previous studies used metal passive implants (i.e., gold) to close the eyelids. In addition to passive mechanisms used in that method and inability to create eyelid movements and/or blinking, the function of that method depended on gravity. To use that method, the muscle levator palpebral superiors must be somewhat sensitive to respond to the corneal stimulation [51]. This means that method could not move the eyelids with fully inactive muscles. On the other hand, in some other treatment methods such as temporalis transfer or hypoglossal transposition, the patients must be able to perform minor actions such as masticate or protrude the tongue to extend the face skin, produce the desired extension/movement, and then consequently produce corneal reflex [52]. However, the mechanisms of model A, and specifically B, in the present study do not depend on gravity and can work without needing any sensitivities, activations, or even minor actions from any muscles. 

The results showed that in model A, the risk of mechanical stress damage like stress concentration in IPMC with metallic electrodes was at least 98.5% higher than those of in nanocomposite electrodes in a similar condition (Figure 6b,f). The corresponding risk of mechanical stress damage for model B was 30.4% (Figure 6d,h). In addition, there are other disadvantages in IPMC with metallic electrodes including less biocompatibility, a higher weight of metallic electrodes (Table 1) as well as a higher risk of inflammation, cracking, and delamination [53,54]. Moreover, the biological fluids as an electrolyte solution can play an anode role for gold and platinum and cause corrosions, oxidation, and less lifespan [55,56]. Therefore, in both models, IPMCs with metallic electrodes cannot be optimal solutions. Hence, we compare the efficiency of IPMC with nanocomposite electrodes in models A and B.

The effective output in model A was the generated force in the tip of IMPC. We set the generated force of IPMC with nanocomposite electrodes in the normal physiological range (0.37–0.55 N [44]). In regard to a study by Tollefson [16], an actuator with less generated force (IPMC with nanocomposite electrodes) can be an option that is more effective. IPMC with BSA–CNT has 25.4% lower force as compared to IPMC with MCC–CNT electrode. The risk of mechanical stress damage like stress concentration in IPMC with MCC-CNT electrode was 83.0% less than in IPMC with BSA-CNT. However, model A, even with IPMC with nanocomposite electrodes, has limitations and issues compared to model B for efficient movement of the eyelids and blinking. In model A, a sling is connected with the IPMC actuator to create blinking. In regard to the use of a sling, more voltage and force are needed due to the elasticity and friction of the sling material [15] on the lateral orbital edge, while in model B, IPMC can work directly without any intermediates, such as using a sling to blink. Another concern for use in model A is the gravity effect. Model B is free from the gravity effect and active IPMC is able to move the eyelids directly and independently. Another common issue of model A in previous studies was its problem with movement of the lower eyelid as compared to the upper eyelid, and its ability to make a non-symmetric movement [16], while model B is able to move the lower eyelid independently like the upper eyelid to make a symmetric movement. The results of a study by Hontanilla et al. showed that model A may also accompany malposition issues [52]. Therefore, it can be deduced that model B can better meet the medical and mechanobiological considerations without the concerns related to model A. 

In model B, the efficiency of IPMC with MCC–CNT electrodes in producing the higher linear displacement (the effectiveness output in model B) and lower mechanical stress damage was 77.0% and 77.1% higher than IPMC with BSA–CNT electrodes. IPMC with MCC–CNT was able to create an acceptable angular displacement (−38.28° to 41.14°) to create normal blinking since we designed the initial position of IPMC in the middle of each eyelid. Therefore, IPMC with an MCC–CNT electrode can be a more efficient option to use in model B. It should be noted that there were no considerable differences (<1%) between the cation concentration values in model B with different electrodes (Figure 8b). This can be related to the use of a common membrane (Nafion layer) in IPMC with different electrodes. It is worth mentioning that in addition to the abovementioned issues with the IPMCs with metallic electrodes, the efficiency of IPMCs with metallic electrodes in producing higher displacements was also much less than IPMCs with nanocomposite electrodes in this model. Another important parameter for opening/closing the eyelids is the frequency of blinking. According to a study by Akamatsu [57], normal blinking occurs 5 to 20 times per 60 s (each blink occurs in 3–12 s). The results in Figure 6c showed that IPMC with MCC–CNT electrodes in model B can create each blink in 4 s, which is in the normal range. This result confirms the acceptable sharpness of the model B mechanism to make blinking and the correctness of the frequency (π/4) of input voltage. 

Taken together, IPMC with MCC–CNT electrodes in model B were the most efficient options to restore the eyelid movements in patients with ptosis. The assessment of the effect of the rolled graphene layers on nanocomposites is also of great importance [58,59,60]. Table 2 shows the changes in electrical properties of MCC–CNT electrodes in different weight percentages and numbers of CNT under two rolled graphene layers: multi-wall CNT (MWCNT) and single-wall CNT (SWCNT). The corresponding values for BSA are listed in Supplementary Information (Table S1). The results of Figures S1 and S2 show the weight percentages of CNT to achieve the optimal electrical conductivity (1.31 × 10^−6^ S/m), in MCC–MWCNT it was 0.110% and MCC–SWCNT it was 0.016%. In these optimal percentages, the differences in calculated results (displacements, stress, and cation concentration) between MCC–MWCNT and MCC–SWCNT were not considerable. However, MCC–SWCNT achieved the optimal electrical conductivity in a lower weight percentage of CNT. It should be noted that the results of a study by Gerasimenko et al. showed that polymer-based SWCNT nanocomposites are not an adequately acceptable option for biological applications due to their higher toxicity risk [26].

### Limitations and Future Prospects

A battery with wire was utilized to supply the voltage of the actuator in previous studies (Figure 1a,b) [9,15,16]. A wireless battery with the microchip antenna can be installed with IPMC actuators as an alternative, as compared to the previous voltage supplement system (battery and wires). Due to short distances between battery and actuators, and simultaneous and uniform action of all actuators in blinking, the use of the wireless battery can be assessed as an appropriate alternative in future studies. It should be noted that the eyelid implant created on the material considered in this paper will consume negligible energy (<10 J/day). Therefore, the battery located under the skin will be enough to charge once for 1–2 months using known wireless power transmission systems [61]. In addition, future studies can consider the electromyography (EMG) signal of the healthy eyelid muscles [62] in the definition of the input voltage of IPMC. In this case, to make the unique blinking in patients with unilateral ptosis (such as some types of hydrocephalus patients [63]), the movement of an eyelid with the implanted actuator can be similar to another healthy eyelid of that eye. In addition, it is suggested for future studies to create the suggested enhanced IPMC with a MCC electrode under model B for restoring eyelid movement. Previous studies can help create the model [64,65]. For a better illustration of the function of this model, it can be implemented in an animal or cadaver experiment. It should be noted that CNTs may be aggregated and clustered, hence, this can be a challenge in creating and implementing the model.

## 5. Conclusions

In the present study, the enhanced IPMCs with nanocomposite electrodes were used under two different mechanisms as an artificial muscle to restore eyelid movement in patients with ptosis. In the mechanism of model A, IPMC and eyelid muscle were connected through a sling, and model B moved the eyelids directly and independently. In both models A and B, IPMC with metallic electrodes had some drawbacks such as a higher risk of mechanical stress damage, less biocompatibility, a higher weight of metallic electrodes, a higher risk of inflammation, cracking, delamination, corrosions, oxidation, and less lifespan. Despite the higher efficiency of IPMC with nanocomposite electrodes in model A, model A has some limitations in restoring eyelid movements: working under a higher voltage compared to model B, demanding higher force due to elasticity and friction of sling, the possibility of malposition and non-symmetric movement of the lower eyelid. Model B with nanocomposite electrodes was the option that is most efficient. The efficiency of the IPMC with MCC–CNT nanocomposite electrodes under model B in producing higher displacements (both linear and angular) and lower mechanical stress damages was higher than BSA–CNT. Therefore, IPMC with MCC–CNT electrodes under the mechanism of model B can be the most efficient option to restore eyelid movement.

## Figures and Tables

**Table 1:** Material properties of electrodes, membrane, and basic polymer.

Material Properties	Membrane (Nafion)	Platinum	Gold	BSA-CNT	MCC-CNT	CNT	BSA	MCC
Young modulus (Pa)	0.249 × 10^9^	1.58 × 10^11^	7.57× 10^10^	1.2× 10^9^	1.2× 10^8^	10^12^	10^9^	10^8^
Poisson ratio	0.48	0.34	0.44	0.23	0.30	0.3	0.23	0.3
Density (gr/cm^3^)	1.97	2.13	1.92	1.25	1.75	1.7	1.1	1.5
Electrical conductivity (S/m)	-	8.74 × 10^7^	4.44 × 10^7^	1.27 × 10^−6^ *	1.31 × 10^−6^ *	10^7^	10^−14^	10^−10^
Permittivity	-	0.14	1.62	10	2	1200	10	1.85

* The amounts of optimal electrical conductivity of BSA-CNT and MCC-CNT were calculated based on optimal weight percentage of CNT using MATLAB and COMSOL softwares.

**Table 2:** The changes in electric field norm (V/m), current density norm (A/m^2^), and electrical conductivity (S/m) in MCC–CNT with different rolled graphene layers.

MCC–MWCNT				
CNT wt%	CNT Number	Electric Field Norm (V/m)	Current Density Norm (A/m^2^)	Electrical Conductivity (S/m)
0.0099471	9	14,128	2.74E-02	1.94E-08
0.022104	20	14,425	2.74E-02	1.90E-08
0.088399	80	14,815	3.52E-02	2.37E-08
0.11049	100	28,414	9.30E+00	1.31E-06
0.17676	160	30,535	4.3184	1.41E-06
0.19884	180	30,027	3.8108	1.27E-06
0.20988	190	29,287	3.4376	1.17E-06
MCC–SWCNT				
0.0014211	9	14,128	2.74E-02	1.94E-08
0.0031579	20	14,425	2.74E-02	1.90E-08
0.012632	80	14815	3.52E-02	2.37E-08
0.01579	100	28,414	9.30E+00	1.31E-06
0.025263	160	30,535	4.3184	1.41E-06
0.028421	180	30,027	3.8108	1.27E-06
0.03	190	29,287	3.4376	1.17E-06

## Data Availability

Not applicable.

## Supplementary Materials

The following supporting information can be downloaded at: https://www.mdpi.com/article/10.3390/nano13030473/s1, Figure S1: Electrical conductivity in BSA-SWCNT and MCC-SWCNT (0.00–0.05 wt%); Figure S2: Electrical conductivity in BSA-MWCNT and MCC-MWCNT (0.00–0.3 wt%); Table S1: The changes in electric field norm (V/m), current density norm (A/m^2^), and electrical conductivity (S/m) in BSA-CNT electrode with different rolled graphene layers.
