# Supplementary material for: Enhanced Ionic Polymer–Metal Composites with Nanocomposite Electrodes for Restoring Eyelid Movement of Patients with Ptosis
Source: Nanomaterials (Basel). 2023 Jan 24;13(3):473. doi: 10.3390/nano13030473 (PMC9920823; doi:10.3390/nano13030473)
Supplement: Supplementary file 1 [file nanomaterials-13-00473-s001.zip › nanomaterials-2098340-supplementary.pdf]

Supplemental Material

Enhanced Ionic Polymer–Metal Composites with Nanocomposite Electrodes for Restoring Eyelid Movement of Patients with Ptosis

Sara Sadat Hosseini [1], Bakhtiar Yamini [1], Levan Ichkitidze [2,3], Majid Asadi [4], Julie Fernandez [1] and Seifollah Gholampour [1,*]

[1] Department of Neurological Surgery, University of Chicago, Chicago, IL 60637, USA
[2] Institute of Biomedical Systems of National Research University of Electronic Technology (MIET), 124498 Moscow, Russia
[3] Institute of Bionic Technologies and Engineering of I.M. Sechenov First Moscow State Medical University, 119991 Moscow, Russia
[4] Northern Michigan University, Marquette, MI 49855, USA
* Correspondence: seifgholampour@bsd.uchicago.edu

Effect of rolled graphene layers on the BSA-CNT electrode:

The electrical properties of the BSA-CNT electrode include electric field norm (V/m), current density norm (A/m2), and electrical conductivity (S/m) are listed in Table S1 based on the weight percentage and number of CNT in two rolled graphene layers condition: multi-wall CNT (MWCNT) and single-wall CNT (SWCNT). The optimal electrical conductivity of 3.28E-04 is obtained for BSA-SWCNT and BSA-MWCNT in weight percentages of 0.02% and 0.15%, respectively (Figs. S1, S2). In these optimal percentages, the differences in calculated main results (displacements, stress, and cation concentration) between BSA-MWCNT and BSA-SWCNT were not considerable and we did not report them.

CNT wt%	CNT Number	BSA-MWCNT<br>Electric field norm (V/m)	Current density norm (A/m^2)	Electrical conductivity (S/m)
0	0	12000	1.20E-10	1.00E-14
0.013745	9	14128	2.74E-02	1.94E-06
0.030541	20	14425	2.74E-02	1.90E-06
0.12211	80	14815	3.51E-02	2.37E-06
0.15262	100	28408	9.32E+00	3.28E-04
0.24408	160	30528	4.3179	1.41E-04
0.27455	180	30021	3.8112	1.27E-04
0.28978	190	29286	3.438	1.17E-04
		BSA-SWCNT		

0	0	12000	1.20E-10	1.00E-14
0.0019636	9	14128	2.74E-02	1.94E-06
0.0043636	20	14425	2.74E-02	1.90E-06
0.017454	80	14815	3.51E-02	2.37E-06
0.021817	100	28408	9.32E+00	3.28E-04
0.034906	160	30528	4.3179	1.41E-04
0.039269	180	30021	3.8112	1.27E-04
0.04145	190	29286	3.438	1.17E-04

Table S1. The changes in electric field norm (V/m), current density norm (A/m^2), and electrical conductivity (S/m) in BSA-CNT electrode with different rolled graphene layers.
BSA: bovine serum albumin, CNT: carbon nanotube, SWCNT: single-wall CNT, MWCNT: multi-wall CNT.

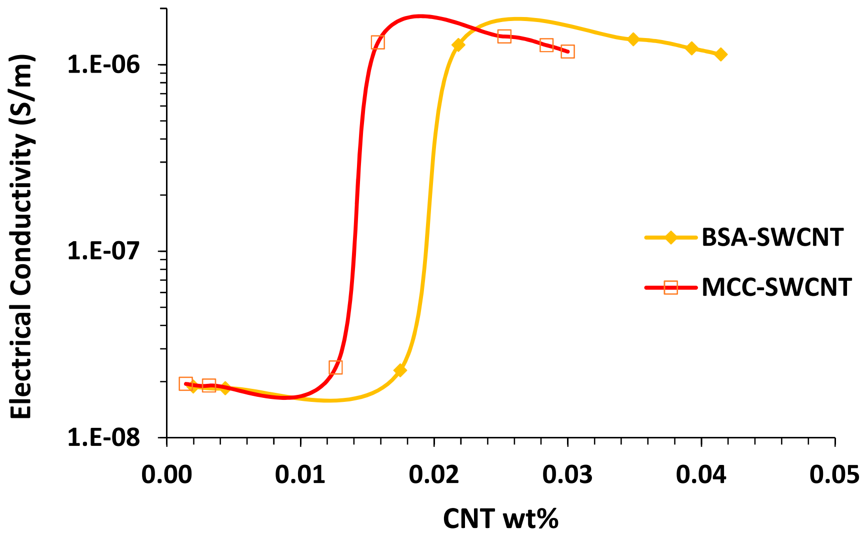

Figure. S1. Electrical conductivity in BSA-SWCNT and MCC-SWCNT (0.00-0.05 wt%).
MCC: microcrystalline cellulose, BSA: bovine serum albumin, CNT: carbon nanotube, SWCNT: single-wall CNT, MWCNT: multi-wall CNT.

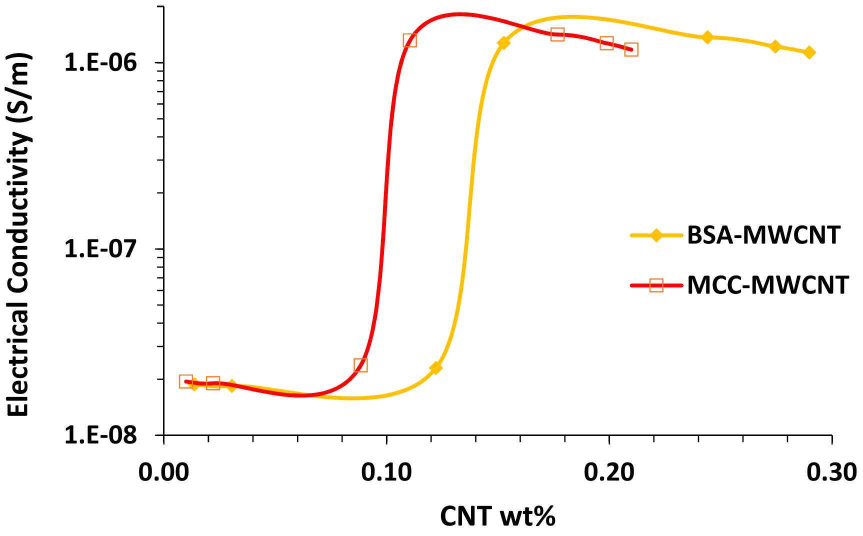

Figure. S2. Electrical conductivity in BSA-MWCNT and MCC-MWCNT (0.00-0.3 wt%). .
MCC: microcrystalline cellulose, BSA: bovine serum albumin, CNT: carbon nanotube, SWCNT: single-wall CNT, MWCNT: multi-wall CNT.
